# Looking Back, Looking Forward: Current Medications and Innovative Potential Medications to Treat Alcohol Use Disorder

**DOI:** 10.35946/arcr.v42.1.11

**Published:** 2022-10-20

**Authors:** Barbara J. Mason

**Affiliations:** Pearson Center for Alcoholism and Addiction Research, Department of Molecular Medicine, Scripps Research Institute, La Jolla, California

**Keywords:** alcohol, alcohol use disorder, treatment, medication, disulfiram, naltrexone, acamprosate

## Abstract

This article is part of a Festschrift commemorating the 50th anniversary of the National Institute on Alcohol Abuse and Alcoholism (NIAAA). Established in 1970, first as part of the National Institute of Mental Health and later as an independent institute of the National Institutes of Health, NIAAA today is the world’s largest funding agency for alcohol research. In addition to its own intramural research program, NIAAA supports the entire spectrum of innovative basic, translational, and clinical research to advance the diagnosis, prevention, and treatment of alcohol use disorder and alcohol-related problems. To celebrate the anniversary, NIAAA hosted a 2-day symposium, “Alcohol Across the Lifespan: 50 Years of Evidence-Based Diagnosis, Prevention, and Treatment Research,” devoted to key topics within the field of alcohol research. This article is based on Dr. Mason’s presentation at the event. NIAAA Director George F. Koob, Ph.D., serves as editor of the Festschrift.

According to national surveys, more than 85% of U.S. adults have consumed alcohol at some point in their lifetime, and about 70% did so in the last year.^1^ Most individuals drink responsibly and without adverse effects. However, a substantial proportion of people misuse alcohol.^1^,^2^ Drinking patterns associated with alcohol misuse include binge drinking, heavy drinking, and high-intensity drinking. Binge drinking is typically defined as five or more drinks within a few hours for men and four or more drinks within a few hours for women. Heavy drinking is defined as four or more drinks per day or more than 14 drinks per week for men and more than three drinks per day or more than seven drinks per week for women. High-intensity drinking is defined as two or more times the gender-specific thresholds per day for binge drinking.^3^ Moreover, according to the National Survey on Drug Use and Health, nearly 15 million people age 12 and older in the United States, or 5% of this age group, met the criteria for alcohol use disorder (AUD) in 2019.^1^ Alcohol misuse and AUD exert a heavy toll on the individual, their families and communities, and society as a whole. Alcohol contributed to about 99,000 deaths in 2020, making it one of the leading preventable causes of death in the United States.^4^ Additionally, alcohol misuse imposes a heavy economic burden on the nation.^5^ Indeed, in many respects, alcohol misuse and its consequences are a substantially greater societal problem than the current opioid crisis, yet it generally receives less attention.^6^–^10^ Therefore, the development and availability of effective treatments for AUD are of utmost importance.

Various treatment approaches have been identified for AUD, including pharmacological and nonpharmacological approaches. However, only a small proportion of people with AUD receive treatment. In 2019, only about 7% to 8% of these individuals were estimated to receive any treatment for AUD, and less than 2% reported using a medication approved by the U.S. Food and Drug Administration (FDA) for the treatment of AUD.^11^ To date, only three medications—disulfiram, naltrexone, and acamprosate—have been approved by FDA for the treatment of AUD. Development of additional medications has largely been ignored by the pharmaceutical industry and instead is being driven by grants from the National Institute on Alcohol Abuse and Alcoholism (NIAAA) to academic scientists, as well as by work conducted by NIAAA’s Clinical Investigations Group and Intramural Research Program. Numerous potential medications—including those currently used for other indications as well as newly developed medications—are being investigated and will hopefully help to increase the number of treatment options available to people with AUD and their health care providers. This article describes the characteristics, benefits, and risks of the FDA-approved medications for AUD; evaluates the benefits and risks of novel drugs repurposed for the treatment of AUD; and appraises novel drug targets that are in the pipeline.

## FDA-Approved Medications

The three FDA-approved drugs to treat AUD have very different mechanisms of action, but they share some key characteristics:

- For AUD medications, the pivotal clinical trials conducted to gain FDA approval involved behavioral counseling for all the participants—both those receiving the active drug and those receiving placebo. In these pivotal trials, drinking outcomes were better for participants who received the medication plus counseling than for those who received placebo plus counseling, demonstrating an incremental benefit of using evidence-based medications combined with counseling beyond that shown by counseling alone.^12^,^13^
- All three medications are not to be used as a stand-alone treatment but as part of a comprehensive treatment plan based on a chronic care model, as suggested in the 2016 *Surgeon General’s Report on Alcohol, Drugs, and Health*.^14^ This comprehensive model includes medications and additional components such as behavioral therapies and recovery support services.
- Like behavioral counseling and other therapeutic approaches, the medications are a treatment for AUD but are not a cure. Moreover, they are not a treatment for alcohol withdrawal, which requires its own special type of medication.
- The medications do not substitute for alcohol—such as methadone does for opioids in the treatment of opioid use disorder—and they do not induce euphoria. Moreover, the drugs are not addictive; people taking them long term do not develop tolerance or experience withdrawal upon treatment discontinuation. Accordingly, these medications have no street value as illicit drugs.^15^

To obtain FDA approval for a medication to treat AUD, manufacturers must assemble a safety dossier that includes studies demonstrating that the medication itself has no misuse potential and does not harmfully interact with alcohol, for example, by further impairing awareness or coordination if a person consumes alcohol while taking the drug. Additionally, the medication must demonstrate efficacy in typically two pivotal trials—defined as randomized, double-blind, placebo-controlled, multicenter trials that ideally represent the four quadrants of the United States and have adequate representation of women and minority participants.^16^ Pivotal trials typically are 6 months in duration, during which the medication is given in conjunction with behavioral counseling. Primary outcome measures used by FDA to determine efficacy generally are rates of days with no drinking or no heavy drinking (i.e., consumption of five or more drinks per day for men and four or more drinks per day for women).^15^ To determine these outcomes, a standard “drink” is defined as the beverage volume that contains 0.6 oz. of pure ethanol, which corresponds to approximately 12 oz. of beer, 5 oz. of wine, or 1.5 oz. of distilled spirits.^17^

To determine participants’ alcohol consumption in trials submitted for FDA approval, self-report instruments such as the Timeline Follow-back Interview capture the self-reported number of daily drinks consumed.^18^ These self-reports are often combined with biochemical measures, such as breathalyzer readings, alcohol glucuronide urine dipsticks, or blood tests for the levels of the liver enzyme gamma-glutamyl transferase (GGT). Breathalyzer analyses only capture alcohol consumption at the time of the study visit whereas the alcohol glucuronide urine dipstick may reflect drinking up to 3 days before the study visit. Blood levels of GGT are an indirect marker of more distant alcohol consumption; they typically rise after heavy alcohol consumption that has continued for several weeks and may have occurred as long as 3 weeks prior to the study visit.^19^ Smartphone apps for real-time drinking data collection and noninvasive transdermal wrist alcohol sensors are under development, but have not been used in regulatory studies.

### Disulfiram

The first medication to be approved for AUD was disulfiram (Antabuse, now available in generic formulations), which entered the market in 1951—thus preceding even the inception of NIAAA. Its mechanism of action involves inhibition of the enzyme aldehyde dehydrogenase, which plays a central role in alcohol metabolism, converting the alcohol metabolite acetaldehyde into acetate. If an individual consumes alcohol while having disulfiram in their system, the drug will inhibit the acetaldehyde metabolism, resulting in rapid acetaldehyde accumulation that leads to a quick onset of flushing, nausea, palpitations, and other symptoms that can become quite severe and at times life-threatening. This mechanism of action acts as a psychological deterrent to any alcohol use.

Because of the rapid and potentially even fatal consequences of acetaldehyde accumulation after taking disulfiram, the medication should never be given to individuals with acute alcohol intoxication or without their full knowledge.^20^ Additionally, individuals should be instructed to abstain from alcohol for at least 12 hours before taking disulfiram and be advised that reactions with alcohol can occur for as long as 14 days after discontinuing the medication. Disulfiram itself is associated with some hepatotoxicity; therefore, the patient’s liver function should be measured before and during disulfiram treatment to ensure safety.

Disulfiram can only exert its effects if taken regularly. Studies found that outcomes are better in patients with high medication adherence who are strongly motivated to quit drinking, as well as in those patients who have a partner who is supportive of their recovery and will supervise the daily administration of disulfiram.^21^,^22^

### Naltrexone

FDA originally approved naltrexone in 1984 for opioid addiction; for treatment of AUD, it was approved as an oral medication in 1994 (Revia and generic formulations) and as a long-acting injectable medication in 2006 (Vivitrol).^23^–^25^ Naltrexone is a pure mu-opioid receptor antagonist that binds to the receptor, thereby blocking some of the rewarding effects of alcohol. Blunting the rewarding effects of drinking is thought to reduce alcohol consumption and thereby promote recovery from AUD. Consistent with this hypothesis, meta-analyses of numerous naltrexone studies have shown that, compared to placebo, the medication decreases heavy drinking.^12^,^13^

As with disulfiram, the efficacy of naltrexone is affected by adherence problems associated with oral dosing. To address these problems, a once-monthly, extended-release, intramuscular injection formulation has been developed.^25^ Because naltrexone has such a specific mechanism of action, researchers have attempted to identify genetic predictors to determine which individuals with AUD might be most likely to respond to naltrexone treatment. To date, these investigations have not yielded reliable results; for example, initial findings of an association of different variants of the mu-opioid receptor gene *OPRM1* with response to naltrexone could not be replicated in other clinical trials.^26^ However, the investigations into genetic predictors of naltrexone response are still ongoing.

Naltrexone treatment is generally considered safe. But because of the medication’s effects on the mu-opioid receptor, it is important to rule out co-occurring illicit or prescribed opiate use prior to initiating treatment for AUD to avoid inducing acute opioid withdrawal. Also, like disulfiram, naltrexone itself is associated with some hepatotoxicity, particularly in higher doses. However, because alcohol itself is a hepatotoxin, naltrexone actually can improve liver functioning by reducing alcohol intake in individuals with AUD.^23^

### Acamprosate

Disulfiram and naltrexone both aim to reduce drinking by making alcohol consumption a less pleasant experience, either by causing unpleasant effects after alcohol consumption or by reducing alcohol’s stimulation of receptors in the brain’s reward system. Acamprosate (Campral) takes a different strategy that is based on the observation that heavy drinking and withdrawal dysregulate the balance between the excitatory (glutamatergic) and inhibitory (primarily gamma-aminobutyric acid [GABA]-ergic) neurotransmitter systems in the brain. In particular, the excitatory system becomes hyperactive during early abstinence, and acamprosate has been shown to restore homeostasis in this system by reestablishing normal *N*-methyl-D-aspartate receptor tone in the glutamate system.^27^,^28^

Clinical studies lasting up to 1 year found that acamprosate treatment increased rates of abstinence relative to placebo.^12^,^15^ Post-treatment follow-up studies found that these effects persisted for as long as 1 year after the last dose of medication.^12^,^15^,^28^ Thus, unlike disulfiram and naltrexone, acamprosate seems to restore normal function in aspects of the brain’s glutamatergic signaling system that can result in long-term AUD treatment effects. One additional component contributing to acamprosate’s effectiveness may be its normalizing influence on alcohol-related sleep disturbances, which can be quite severe in early abstinence and precipitate relapse to drinking.^29^,^30^

Researchers at the Mayo Clinic have been seeking to identify predictors of patient response to acamprosate. They determined that a patient’s serum glutamate concentrations at baseline could serve as a biomarker of treatment outcome, with high serum glutamate levels predicting a good response. Moreover, individuals who showed a response to acamprosate treatment showed the greatest reduction in serum glutamate levels from baseline to the end of treatment.^31^

Unlike disulfiram and naltrexone, acamprosate is not metabolized in the liver. Therefore, it is safe to use in patients with hepatic impairment. However, as acamprosate is excreted through the kidneys, it is important to ensure that patients do not have severe renal impairment. Acamprosate has low bioavailability, which necessitates that the medication be taken three times per day.^15^

### U.S. Treatment Guidelines

In 2017, the American Psychiatric Association issued practice guidelines for the pharmacological treatment of patients with moderate to severe AUD.^20^ These guidelines recommend use of acamprosate or naltrexone in patients who wish to cut down or quit drinking, who prefer medication or have not responded to nonpharmacological treatments, and who have no medical contraindications to use of these drugs. Disulfiram is not recommended as a first-line treatment for AUD, given (a) the potential risk of severe reactions and physiological consequences of drinking while taking the drug, and (b) the more robust evidence for efficacy in acamprosate and naltrexone. However, it may be used in patients who prefer disulfiram or are intolerant to or have not responded to naltrexone or acamprosate and who understand the risks of alcohol consumption while taking disulfiram.

The guidelines also recommend that antidepressant medications and benzodiazepines should not be used for the treatment of AUD unless the individual has been diagnosed with a concurrent disorder (e.g., depression, anxiety) for which these medications are indicated. Benzodiazepines can be used to manage acute alcohol withdrawal for up to 5 days; beyond that time, there is no support for the use of benzodiazepines in the treatment of AUD, especially because benzodiazepines themselves have misuse potential and are cross-tolerant with alcohol.^20^

### Nalmefene—Widely Approved Outside the United States

A fourth medication, nalmefene, has been approved for treatment of AUD throughout the European Union, the United Kingdom, and other countries. In contrast to naltrexone, which mainly binds to the mu opioid receptor, nalmefene acts as a more potent antagonist at the mu, delta, and kappa opioid receptors.^32^ Nalmefene’s activity at the kappa opioid receptor is of interest because activation of this receptor is associated with increases in anxiety and dysphoria. Consequently, by blocking this array of receptors, nalmefene may diminish both the rewarding effects of alcohol as well as the anxiety and dysphoria associated with not drinking in individuals with AUD.

In contrast to oral naltrexone, disulfiram, and acamprosate, which must be taken daily, nalmefene is only taken 1 to 2 hours prior to anticipated drinking occasions. Follow-up studies of up to 1 year found that nalmefene treatment was associated with decreased drinking relative to placebo.^33^–^35^ The European Medicine Agency based its approval on slightly different treatment outcome criteria than FDA, including a two-level reduction in World Health Organization (WHO) drinking risk levels.^36^ (These risk levels—very high, high, medium, and low—are defined based on estimated mean daily ethanol consumption in grams in the previous 12 months.^37^) This level of reduction in alcohol consumption has been shown to be associated with improved mental health, particularly anxiety and depression; improved physical health (liver functioning); and improved overall quality of life.^38^ Thus, both the FDA-approved drinking outcomes (i.e., rates of no drinking or no heavy drinking days) and the WHO risk level-based outcomes have broad clinical relevance for individuals with AUD.

## Identifying New Medications for AUD

**Figure 1:**
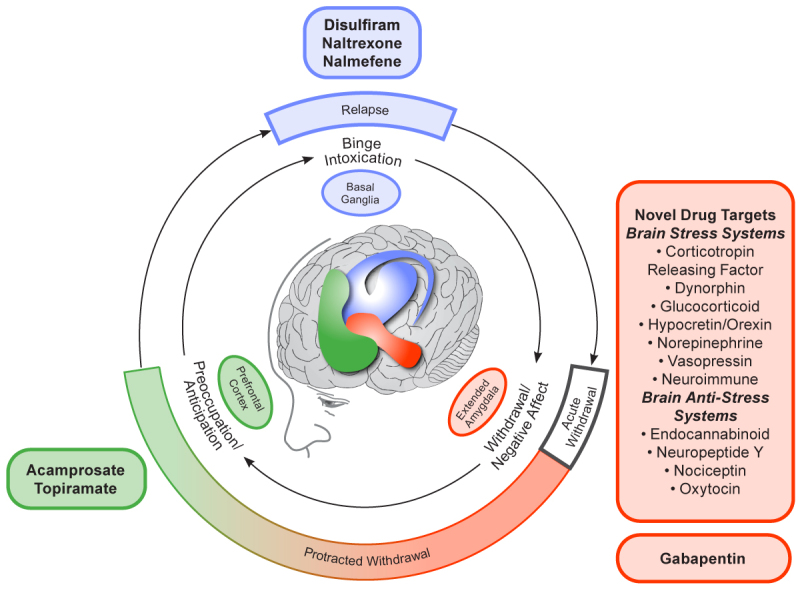
Conceptual framework for the effects of various medications on the three major stages of the alcohol addiction cycle and the clinical stages of alcohol use disorder The outer ring relates to the clinical stages of alcohol use disorder; the inner ring relates to the three stages of the addiction cycle. *Note:* Adapted by permission from Springer Nature: *Neuropharmacology,* 35(1):217–238. Neurocircuitry of addiction. George F. Koob and Nora D. Volkow, 2010.^59^

As described above, the currently approved medications for AUD largely focus on reducing the expected positive or rewarding effects of drinking. Disulfiram, naltrexone, and nalmefene all interrupt the binge intoxication phase, either by inducing negative responses after drinking or by reducing alcohol’s rewarding effects, whereas acamprosate reduces craving in the preoccupation-anticipation phase in individuals in recovery (see Figure 1). Use of these anti-reward medications is based on the assumption that most people drink due to positive reinforcement—they want to experience alcohol’s rewarding effects—and that taking away those effects will thereby discourage further drinking. However, as recent research has uncovered more information on alcohol’s effects on the brain and the neurobiology of AUD, it has become clear that many people drink because of negative reinforcement—they want to avoid the negative effects of not drinking.^39^ In people with AUD, drinking cessation acts as a stressor because the brain has become used to the presence of certain alcohol levels. In these individuals, abstinence induces excessive activation of the brain stress systems, particularly in the central nucleus of the extended amygdala. This stress response includes the release of corticotropin-releasing factor (CRF), especially in early abstinence, which prompts symptoms of anxiety, dysphoria, irritability, and sleep disturbance that are characteristic of post-acute or protracted withdrawal.^39^ At the same time, the brain’s anti-stress systems often are depleted in early abstinence.^39^ These observations have launched a new avenue of research in the quest for effective treatments for AUD focusing on the neuropeptides that have been shown to be dysregulated during early abstinence and which are associated with the symptoms of protracted withdrawal as potential drug targets for novel medications. The hope is that such treatments could interrupt the AUD cycle before reaching the craving and relapse/binge intoxication stage. Building on recent discoveries of the neurobiology of AUD—and particularly the role that the stress response has during early abstinence in driving the AUD cycle toward relapse—the goal is to normalize those systems to support recovery in a way that is safe and acceptable to patients.

To develop and study the effects of such medications, researchers, with support from NIAAA, developed parallel animal and human laboratory models of risk factors for relapse in protracted withdrawal.^40^ The human model employed techniques already used for other purposes in the field. For example, the investigators used affective priming to induce some of the internal risk factors for relapse, such as the affective states mentioned above, by showing participants images associated with those mood states. Additionally, participants were exposed to external risk factors for relapse through visual and olfactory alcohol cue exposure (i.e., they were asked to view and smell a glass of their favorite alcoholic beverage but not drink it). The studies recruited non-treatment-seeking men and women with AUD who were required to remain abstinent for 3 days prior to testing while taking double-blind study medication. Thus, the volunteers were beginning to exhibit an activated stress response and were highly likely to be responsive to the alcohol beverage cues. The main goal of the experiments was to screen medications aimed at reducing the stress response associated with relapse risk in protracted withdrawal. The study participants were randomly assigned to the medication under investigation or placebo for a relatively short dosing period, based on the period needed to achieve steady-state or maintenance dosing, which is typically between 1 and 2 weeks. Participants were then tested on the last day of dosing, using both subjective and objective measures of responsivity to alcohol as well as extensive analyses to evaluate how well tolerated and safe the studied drug was in individuals with AUD. This approach has been used to evaluate the effectiveness of several medication candidates.

### Gabapentin

The first medication studied using this model was gabapentin, an oral anticonvulsant approved by FDA for the treatment of epilepsy and neuropathic pain. It acts by modulating GABAergic activity on voltage-gated calcium channels, which reduces postsynaptic excitability and decreases the release of excitatory neurotransmitters.^41^ Because this activity also helps restore homeostasis in brain stress systems that become activated in early abstinence, gabapentin seemed to be a promising candidate for treatment of AUD. Moreover, several off-label clinical studies reported beneficial effects of gabapentin on symptoms associated with post-acute protracted withdrawal and risk of relapse, such as dysphoria, anxiety, and insomnia.^41^ In fact, several studies reported that gabapentin was effective for treatment of insomnia, including alcohol-related sleep disturbance, indicating that it decreased stage 1 sleep and arousals while increasing slow-wave sleep and sleep efficiency.^41^,^42^ Like acamprosate, gabapentin is not metabolized in the liver and has an acceptable safety and tolerability profile, further supporting its investigation in the treatment of AUD.^41^

To assess the efficacy of gabapentin in the treatment of AUD, Mason and colleagues conducted a human laboratory study in which they randomly assigned 33 volunteers with AUD to receive either 7 days of gabapentin (1,200 mg/d) or placebo and then tested them on the last day of dosing.^43^ These analyses found that participants who were treated with gabapentin had significantly less craving, lower impulse to drink, and less feelings of loss of control over drinking than those who had received placebo. Gabapentin-treated participants also showed benefits compared with placebo across multiple dimensions of sleep, including sleep efficiency, sleep latency, and sleep quality. Moreover, individuals treated with gabapentin did not report next-day dysfunction or somnolence, which often occur after taking sleep medications.

Based on the findings of the initial study, the research team conducted a larger, double-blind, placebo-controlled, dose-ranging clinical trial of gabapentin in 150 outpatients seeking treatment for AUD.^44^ Participants were randomized to 12 weeks of treatment with either the highest FDA-approved dose of gabapentin (1,800 mg/d), the lowest FDA-approved dose (900 mg/day), or placebo. All patients also received weekly abstinence-oriented counseling over the treatment period. Outcomes analyzed include rates of complete abstinence and no heavy drinking; drinking quantity and frequency; GGT levels as an objective indicator of recent alcohol use; as well as measures of craving, sleep disturbance, and negative affective symptoms.

Over the 12-week treatment period, participants who had received the highest dose of gabapentin had significantly less relapse to drinking and higher rates of complete abstinence compared with placebo; relapse and abstinence levels for participants treated with the 900 mg dose were intermediate. Similarly, participants receiving the high gabapentin dose had the highest proportion of individuals with no heavy drinking at about half the sample, which was about twice as much as among participants receiving placebo; the rate of heavy drinking in participants receiving the lower dose of gabapentin was again intermediate. Thus, both of these key outcomes showed significant linear dose effects. Similar results also were observed for quantity and frequency measures of drinking.^44^

Treatment with the 1,800 mg gabapentin dose also yielded the greatest effect on symptoms of protracted abstinence. Participants who had received this dose showed the greatest reduction in negative affective symptoms on the Beck Depression Inventory II; in craving as determined using the Alcohol Craving Questionnaire; and in sleep complaints as measured using the Pittsburgh Sleep Quality Index.^44^ These results replicated the findings obtained in the earlier laboratory study.^42^ Finally, gabapentin treatment was associated with significant reductions in GGT levels, indicating reduced recent alcohol use.^44^ Together, the results supported the conclusion that gabapentin dose-dependently and significantly improved various parameters of AUD, including rates of complete abstinence and no heavy drinking; drinking quantity and frequency; as well as protracted withdrawal symptoms such as craving, sleep disturbance, and negative affect.

Gabapentin was well tolerated, with no serious or unexpected drug-related adverse events or evidence of misuse potential.^44^ To date, numerous studies conducted in the United States and elsewhere have found no evidence of misuse potential for gabapentin in the treatment of AUD.^41^ Bisaga and Evans demonstrated that gabapentin does not interact pharmacokinetically or pharmacodynamically with alcohol.^45^ However, there have been reports that gabapentinoids, such as gabapentin and the newer drug pregabalin, have been misused by people with opioid use disorder who are in withdrawal, people who misuse prescription drugs recreationally, and people who are incarcerated, with self-administered doses greatly exceeding recommended doses.^41^ Heightened monitoring for gabapentin misuse is warranted in these at-risk populations.

Based on these and other studies supporting the efficacy of gabapentin in treating AUD, the American Psychiatric Association has included gabapentin and another anticonvulsant, topiramate, in its 2017 *Practice Guideline for the Pharmacological Treatment of Patients With Alcohol Use Disorder*.^20^ These guidelines recommend that gabapentin or topiramate be used in patients who have a goal of decreasing or quitting drinking, who prefer gabapentin or topiramate, who are intolerant to or have not responded to acamprosate or naltrexone, and who have no contraindications to the use of gabapentin or topiramate.

### Mifepristone

Mifepristone is a medication approved by FDA for Cushing’s syndrome that shows promise as a repurposed medication for the treatment of AUD and acts upon the protracted withdrawal phase of the AUD cycle.^46^ Mifepristone is a mixed glucocorticoid/progesterone receptor antagonist that has been hypothesized to normalize the altered activity of the hypothalamic-pituitary-adrenal axis. As previously mentioned, heavy alcohol consumption and subsequent withdrawal have an impact on the body’s stress response, with heavy alcohol use blunting activity of the hypothalamic-pituitary-adrenal axis and the stress associated with subsequent abstinence driving CRF release in the amygdala, which contributes to protracted withdrawal symptoms.^47^,^48^ Consequently, administration of mifepristone to people with AUD following acute withdrawal was hypothesized to normalize the individual’s stress response and CRF dysregulation, thereby protecting against relapse during protracted withdrawal.

To investigate this hypothesis, Vendruscolo and colleagues evaluated the effects of mifepristone on people with AUD in a laboratory model of risk factors for relapse.^46^ Participants were randomly assigned to mifepristone (600 mg/d) or matched placebo for 7 days, with testing conducted on the last day of dosing. The analyses found that participants treated with mifepristone not only exhibited significant reductions in craving and response to alcohol cues, but they also had significantly lower levels of drinking than did those who had received placebo, both during the week of treatment and at 1-week follow-up. Moreover, participants receiving mifepristone showed a significant reduction from baseline in GGT levels, the marker of liver function that is sensitive to alcohol intake, as well as in two other measures of liver function (i.e., aspartate transaminase and alanine transaminase levels).^46^

## Looking Ahead in Medication Development for AUD

The clinical studies of both gabapentin and mifepristone showed reductions in craving and alcohol consumption, as well as improvements in liver function tests compared to placebo, suggesting that both medications have therapeutic potential for AUD.^43^,^44^,^46^ Additionally, both medications were well tolerated and triggered no concerns regarding safety, medication adherence, or misuse potential, including rebound craving or alcohol use after medication discontinuation, in these samples of individuals with AUD. These findings provide clinical validation of earlier preclinical studies of protracted withdrawal, in which the medications reduced reinstatement of ethanol seeking and ethanol intake. They also lend support to the role that medications targeting abstinence-related dysregulation in brain stress systems can play as a novel treatment approach for AUD. Such medications may reduce the negative affect and insomnia associated with early abstinence and thereby both increase medication adherence and reduce the misery of early abstinence.

In addition to these studies, which were largely funded by NIAAA, the institute itself has an active research program. The NIAAA Clinical Investigations Group conducts multicenter trials that have shown positive results for two medications relevant to protracted abstinence—varenicline (Chantix), a partial alpha(4)beta(2) nicotinic acetylcholine receptor agonist that FDA has approved as a treatment for smoking cessation, and an investigational vasopressin antagonist.^49^,^50^ The NIAAA Intramural Research Program also has been active in studying molecules that may be relevant to treating protracted abstinence, including ghrelin receptor antagonists^51^ and mineralocorticoid receptor antagonists,^52^,^53^ and a molecule that may show efficacy for both AUD and alcohol-associated liver disease.^54^ These are just a few examples of the many hundreds of drugs that have been evaluated, with NIAAA support, for efficacy in the treatment of AUD.

What characteristics should medications to treat AUD have? Ideally, they should be small molecules that can cross the blood-brain barrier and target the brain regions and systems that are dysregulated by chronic heavy alcohol consumption in a way that is relevant to treating AUD. They should not have misuse potential, nor should they interact with alcohol. This is important so that, in case of relapse, the medication does not exacerbate alcohol’s effects, such as impaired alertness and motor coordination. Additionally, medication candidates should have a good safety profile, particularly no hepatotoxicity; they should show good tolerability with only mild to moderate adverse events that do not prompt treatment discontinuation; they should have good patient acceptability in terms of the route of administration, which is typically oral; and the dosing regimen should be acceptable to the patients and adaptive to their lifestyle.

Another consideration in developing medications for AUD is the potential for sex differences as well as racial differences in drug metabolism, as exemplified by a greater prevalence of the flushing response in certain East Asian populations. Such differences in drug metabolism may affect drug efficacy or safety. Therefore, it is important to have diversity, equity, and inclusion among participants in clinical trials of medications to treat AUD. Sex differences have not been studied systematically for disulfiram and naltrexone; however, although sex does not affect the pharmacokinetics of the long-acting formulation of naltrexone, only men responded to the medication in the pivotal trial, whereas women did not.^25^ The reasons underlying these differences are not fully understood. Sex differences have been comprehensively analyzed for acamprosate in a meta-analysis of individual records obtained from more than 1,300 women and nearly 4,800 men who participated in 22 acamprosate clinical trials.^55^ The meta-analysis found a significant effect of acamprosate relative to placebo on both rates of abstinence and rates of no heavy drinking, and these effect sizes did not differ between men and women. Similarly, the side effect and tolerability profile of acamprosate, including the number, type, and severity of adverse events, did not differ between men and women. Moreover, despite a history of significantly more anxiety, depression, suicide attempts, drug misuse, interpersonal loss, and greater liver impairment at baseline in women than in men, women responded comparably well to acamprosate treatment of AUD.^55^

Another issue to consider in AUD treatment is the age of the patient, as it is never too early (or too late) to treat AUD. In the United States, drinking is illegal for people under age 21, although some in this age group do meet the criteria for AUD. A small, double-blind, placebo-controlled clinical trial of disulfiram conducted in teens found good tolerability and higher rates of abstinence with the medication compared to placebo.^56^ At the other end of the age spectrum, some people older than age 65 have been included in some trials of disulfiram,^57^ naltrexone,^25^ and acamprosate;^58^ however, the numbers were not sufficient to analyze differences in safety and efficacy from younger patients. AUD is a serious concern in older adults because some of its common effects may have more serious consequences in this population, such as the increased risk of falls. Closer monitoring with medication treatment may be necessary if an older person is at increased risk for liver, kidney, or cardiac problems, or uses additional medications to treat other disorders; however, unless there is a medical contraindication, medication treatment is indicated in this age group.

Overall, however, it is clear that new medications to treat people with AUD are urgently needed and that the use of the existing medications must be significantly expanded to support people recovering from AUD. Effective treatments for AUD—both pharmacological and nonpharmacological—are available, but they can only help if they are actually being used. The fact that only 7% to 8% of individuals with AUD report receiving any treatment is a clear indication that much remains to be done in this respect. To support both patients and treatment providers in ensuring that people with AUD receive the appropriate care, NIAAA has created the *NIAAA Alcohol Treatment Navigator* (www.alcoholtreatment.niaaa.nih.gov). This online tool outlines the features of evidence-based AUD treatment, describes the varied routes to recovery, and provides a strategy to help people find practitioners in their area that provide evidence-based treatments, whether behavioral or pharmacological, for AUD.
